# Immunogenicity and safety of concomitant administration of the chinese inactivated poliovirus vaccine with the diphtheria-tetanus-acellular pertussis (DTaP) vaccine in children: A multicenter, randomized, non-inferiority, controlled trial

**DOI:** 10.3389/fimmu.2022.905634

**Published:** 2022-07-26

**Authors:** Xiang Sun, Yan Xu, Fenyang Tang, Yanhui Xiao, Zhiguo Wang, Binbing Wang, Xiaoping Zhu, Xiaoming Yang, Haiping Chen

**Affiliations:** ^1^ Expanded Program on Immunization, Jiangsu Provincial Center for Disease Control and Prevention, Jiangsu, China; ^2^ Medical Affairs, China National Biotec Group Company Limited, Beijing, China; ^3^ Expanded Program on Immunization, Anhui Provincial Center for Disease Control and Prevention, Anhui, China; ^4^ Expanded Program on Immunization, Sichuan Provincial Center for Disease Control and Prevention, Sichuan, China

**Keywords:** sIPV, DTaP, concomitant administration, vaccine interference, safety, immunogenicity

## Abstract

**Key point:**

Considering that vaccination with the sIPV and DTaP overlap at the ages of 3 and 4 months in China, to reduce the burden of treatment on parents and increase vaccination coverage rates, we designed a postmarket clinical study of co-administration.

**Background:**

The Sabin-strain-based inactivated poliovirus vaccine (sIPV) and the diphtheria-tetanus-acellular pertussis vaccine (DTaP) have been licensed in China for many years. To conduct a clinical study on the safety and immunogenicity of the sIPV when administered concomitantly with the DTaP.

**Methods:**

The study population was divided into three groups: group 1 was the sIPV+ DTaP concomitant administration group, group 2 was the sIPV inoculation group, and group 3 was the DTaP inoculation group. Blood samples were collected prevaccination and 30 days postvaccination, and serum antibody levels were detected.

**Results:**

This study showed that the seropositive and seroconversion rates of type 1, 2 and 3 poliovirus in group 1 were higher than those in group 2, with no statistically significant difference after vaccination (P>0.05). Groups 1 and 3 also showed similar responses for all vaccine antigens except anti-FHA (97.65 (94.09-99.36) vs. 100 (97.89-100)). The geometric mean titers (GMTs) for the DTaP and sIPV among the groups were comparable, and the non-inferiority t test result was P<0.001. The number of local adverse events (AEs) reported in group 1 (29.91%) were larger than those in group 2 (12.39%) and group 3 (21.93%), among which the most common was redness. Similarly, the most common systemic AE was fever. All 5 severe AE (SAE) cases were determined by experts to be unrelated to the vaccines during the study.

**Conclusions:**

The evidence of similar seroconversion and safety with co-administered DTaP and sIPV supports the co-administration supports the introduction of a strategy of simultaneous administration of both vaccines into routine infant immunization, and it could increase vaccination coverage and protect more infants from morbidity and mortality from these related diseases.

**Clinical Trial Registration:**

https://clinicaltrials.gov/ct2/show/NCT04054882?term=NCT04054882&cntry=CN&draw=2&rank=1, identifier NCT04054882.

## Introduction

Since the implementation of planned children’s immunization programs in various countries around the world, remarkable achievements have been made in the prevention of diseases through vaccination. The introduction of new vaccines had prevented infectious disease transmission, but the challenge of too many vaccinations has emerged (1). According to the schedule of the childhood immunization program in China, children should receive at least 19 free vaccinations before the age of 3 (2), and the number of vaccinations greatly increases when other self-paid vaccines are added to the schedule. To reduce the frequency of vaccination and achieve higher compliance and better coverage, it is imperative to promote combination vaccines along with simultaneous administration. At present, there are relatively few types of combined vaccines worldwide, and the cost of research and development is high, imposing a heavy burden on some developing countries (3). Therefore, the co-administration of existing established vaccines is particularly important.

The sIPV was developed by China National Biotec Group Company Limited (4), and was approved for drug registration by the State Food and Drug Administration on August 23, 2017 [Lot Number: 2017S00352]. It is mainly administered to those aged 2 months and older and in young children and is used to prevent polio caused by poliovirus types I, II, and III. The DTaP was first approved by the CFDA in 1997 [Lot Number: S10970013]. It is mainly administered to children aged 3 months to 6 years to prevent whooping cough, diphtheria, and tetanus. China’s vaccination guidelines recommend that the sIPV and DTaP be administered 14 days apart. The main reasons for this are to prevent inaccurate attribution of vaccine side effects and because of insufficient data on simultaneous vaccination. It is necessary to verify the safety of the co-administration of vaccines and whether co-administration alters the immunogenicity of the vaccines (5).

Considering that vaccination with the sIPV and DTaP overlap at the ages of 3 and 4 months, to reduce the burden of treatment on parents and children imposed by separate vaccination events and improve the efficacy of the vaccines, we designed a postmarket clinical study of concomitant administration of both vaccines. Healthy infants eligible for inclusion were divided into the sIPV and DTaP group, sIPV group and DTaP group. After completion of the basic immunization procedures for the two vaccines, the immunogenicity and safety of the co-administration group and the single inoculation groups were compared.

## Materials and methods

### Study design

A randomized-controlled(randomization 1:1:1), multicenter, open-label trial was conducted in three centers in Sheyang County in Yancheng city, Jiangsu Province; Si County in Suzhou city, Anhui Province; and Jiangyou County in Mianyang city, Sichuan province; to evaluate the safety and immunogenicity of sIPV and DTaP simultaneous administration. The clinical trial was approved by the ethics committees of the Jiangsu Province Center for Disease Control and Prevention, and the trial was conducted in accordance with statutes or regulations regarding the protection of the rights and welfare of human subjects participating in biomedical research, including the Declaration of Helsinki as amended when the trial began in April 2006, Good Clinical Practice (GCP) and International Conference on Harmonization (ICH) guidelines and Chinese GCPs. Written informed consent of the subject’s parents or guardians was obtained before registration. This study is registered at ClinicalTrials.gov (NCT04054882).

**Figure 1:**
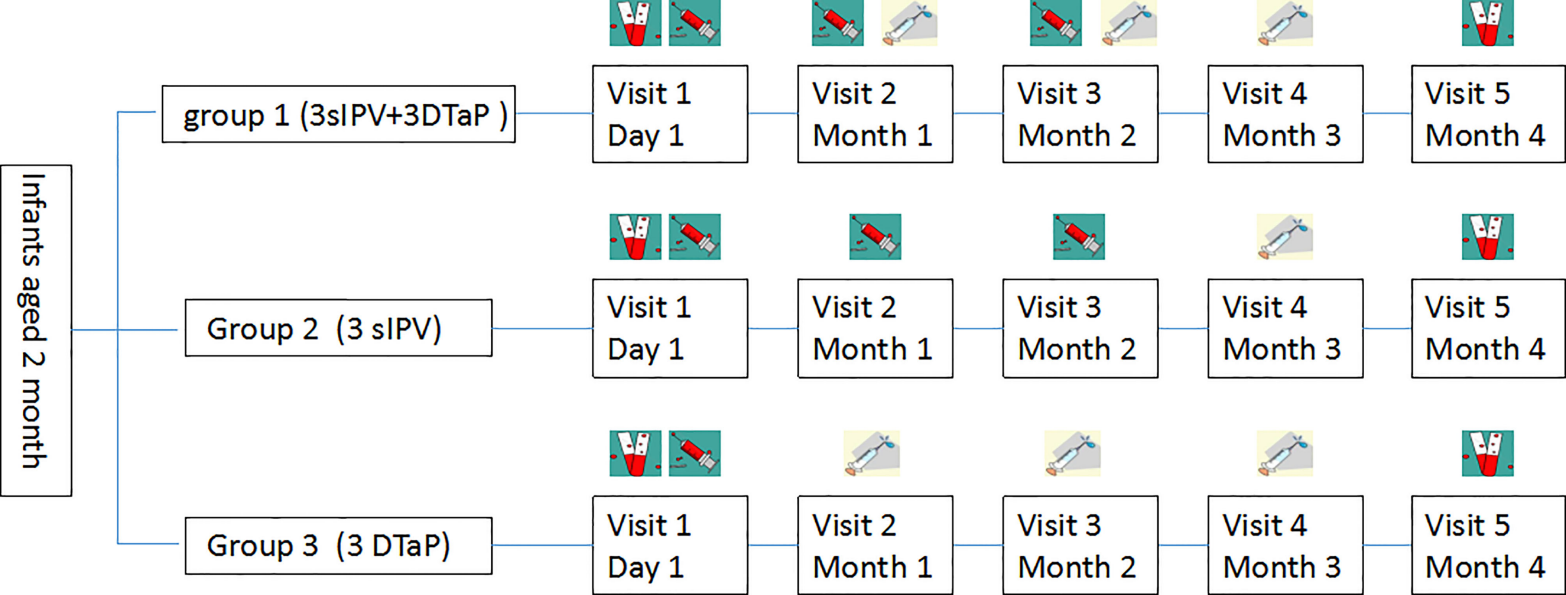
Vaccination schedule in the concomitant and separated groups (
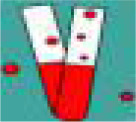
, blood sample;
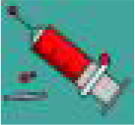
, sIPV;
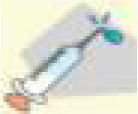
, DTaP.).

The study population was divided into three groups(**Figure 1**): group 1 was the co-administration group, with the first injection of sIPV at 2 months of age; and the simultaneous inoculation of the sIPV (doses 2 and 3) and DTaP (doses 1 and 2) at 3 and 4 months of age, respectively. The third dose of DTaP was administered at the age of 5 months, with an interval of 1 month between each dose. Group 2 was the sIPV group (3 doses of sIPV), with 1 dose of the sIPV administered at 2, 3, and 4 months of age, with an interval of 1 month between each dose. Considering that the national immunization program recommends vaccination with the DTaP at 3, 4 and 5 months of age, the first dose of the DTaP vaccine was administered 7-14 days after sIPV inoculation at 3 months of age, and the second dose of the DTaP vaccine was administered 7-14 days after sIPV inoculation at 4 months of age. The interval between the second and third doses of the DTaP vaccine was 1 month. Group 3 was the DTaP group (3 doses of DTaP), with 1 dose of DTaP was administered at 3, 4, and 5 months of age, with an interval of 1 month between each dose. The subjects in this group were vaccinated with the first dose of the sIPV at the age of 2 months. The second dose of the sIPV was administered 7-14 days after DTaP inoculation at 3 months of age, and the third dose of the sIPV was administered 7-14 days after DTaP inoculation at 4 months of age. The interval between the second and third doses of the sIPV vaccine was 1 month. The immunogenicity purpose of this study was to demonstrate that immune responses to antigens in group 1 were not inferior to those in groups 2 and 3 at one month after vaccination. At the same time, we also assessed the safety of co-administration and the administration of each vaccine alone. Three milliliters of venous blood was collected on the day of vaccination and 30 days after vaccination, and serum antibody levels were detected.

### Participants

The participants in this study were healthy infants aged 60-90 days who had never been vaccinated with an IPV, oral polio vaccine (OPV), DTaP or related vaccines. Before starting the study, their body temperature was confirmed to be ≤37.0°C by clinical examination. The exclusion criteria included (1) a history or family history of severe chronic diseases, allergies, convulsions, epilepsy, encephalopathy or psychosis; (2) allergy to any component of the vaccine; (3) acute fever and infectious disease, with a body temperature of >37.0°C; and (4) any other factor that the investigators believed might influence the study results.

### Randomization and masking

According to the method of block randomization, the research subjects were divided into 78 block groups; each block group had 9 people, for a total of 702 people. The statistical software SAS9.4 was used to ensure that the number of subjects enrolled in group 1, group 2 and group 3 were equal and randomly allocated throughout the trial period. Randomisation lists were completed before enrolment by the Jiangsu Province CDC and given to three participating centers. Group information for each participant was hidden in a separate envelope, and group information is only revealed to investigators and the infant’s parent or legal guardians after registration. The laboratory personnel and biostatisticians do not know the type of vaccine that each group of subjects received.

### Vaccines

The sIPV used in the study (0.5 mL/dose, lot number 201901038) were manufactured by Beijing Institute of Biological Products Co., Ltd., with 15, 45, and 45 D antigen units of poliovirus types 1, 2, and 3, respectively. The DTaP used in the study (0.5 mL/dose, lot number 20181214-1) were manufactured by Chengdu Institute of Biological Products Co., Ltd., and it contained 12.5 limit of flocculation unit of diphtheria toxoid, 3.5 limit of flocculation unit of tetanus toxoid,9 ug protein nitrogen of pertussis protective antigen.

### Immunogenicity assessment

Serum antibodies against sIPV were detected by the serum neutralization method. Poliovirus types 1, 2, and 3 antibody titers ≥ 1:8 were considered positive. The serum positive conversion rate was defined as follows: a titer of neutralizing antibody <1:8 before inoculation, and after inoculation with 3 doses of the vaccine, the antibody titers of poliovirus types 1, 2, and 3 were ≥1:8 (positive) or the neutralizing antibody titer exhibited a four-fold increase. For the DTaP, serum samples were measured by the indirect hemagglutination test (IHA) and are expressed as IU/ml. The titers of anti-pertussis toxoid (PT) and anti-filamentous hemagglutinin (FHA) in serum were determined by ELISA pre- and postvaccination. Seroprotection against diphtheria tetanus was defined as antibody concentrations ≥0.1 IU/ml. If anti-diphtheria ELISA antibody concentrations were <0.1 IU/ml, the Vero-cell neutralization assay was performed for pre- and post-vaccination serum samples (assay cutoff 0.004 IU/ml). Antibody concentrations ≥0.01 IU/ml were considered as protective. Both the ELISA test (antibody concentrations ≥0.1 IU/ml) and Vero-cell test (antibody concentration ≥0.01 IU/ml) were used to define the seroprotection status for the primary endpoint; PT and FHA <20 EU/ml prevaccination and ≥20 EU/ml postvaccination; or a postvaccination increase of at least four times the prevaccination antibody titer (6–8).

### Safety assessment

The patient receiving the vaccine dose was observed for 30 minutes, and a diary card was issued after vaccination. The guardian recorded any AEs within 7 days postvaccination, and serious adverse event (SAE) data were collected by telephone calls within 3 months after full immunization. Local reactions included pain, redness, swelling, induration at the site of inoculation, rash, and pruritus. Systemic reactions included fever, irritability, vomiting, diarrhea, eating disorders, lethargy, and allergic reactions. Any AEs were classified according to guidelines issued by the State Food and Drug Administration, and the researchers determined whether they were related to the vaccine (9).

### Statistical analysis

In this study, the positive conversion rate of neutralizing antibodies in serum after basic vaccination was used as the index for effectiveness evaluation, and the non-inferiority of the positive conversion rate of neutralizing antibodies in the experimental group compared with the control group was tested by statistical methods. The unilateral test α level was 0.025, the efficacy power 1-β was 80%, and the non-inferiority margin was 10%. According to the phase III clinical trial of the IPV vaccine produced by the Beijing Institute of Biological Products Co. Ltd., the seroconversion rates of poliovirus types I, 2, and 3 in the 3-dose IPV trial group after basic immunization were 96.2%, 93.8% and 97.6%, respectively. The minimum seroconversion rate of 93.8% (10) was selected to estimate the sample size, and the maximum sample size required for this study was 172. Considering a 20% drop-out rate, 207 people were included in each group. According to relevant results of the phase IV clinical trial of the DTaP produced by Chengdu Institute of Biological Products Co., Ltd (11)., the seroconversion rates of the three antibodies induced by the DTaP vaccine, PT-Ab, D-Ab and T-Ab, were 97.4%, 100% and 99.5%, respectively. The minimum seroconversion rate was 97.4%, and the final number of participants in each group was 136. Considering the differences in seroconversion rates reported by different studies on the DTaP, the sample size in this study was determined to be 234 people per group.

STATA version 15 was used for statistical analysis. All safety and immunogenicity analyses were descriptive, and Pearson’s chi-square or Fisher’s exact tests and rank-sum tests were used to analyze the results. The geometric mean antibody concentrations/titers (GMCs/GMTs) and seroconversion rates for each vaccine were calculated using their respective 95% confidence intervals (CIs). The geometric means of antibodies among groups were compared using the analysis of variance after logarithmic conversion, and the difference was statistically significant if P ≤ 0.05. Non-inferiority test results were considered significant when the P value <0.05. According to the Chinese Regulatory Agency, non-inferiority was defined as met if the upper limit of the 95% CI for the rate difference was ≤10%.

## Results

### Study participants

**Figure 2:**
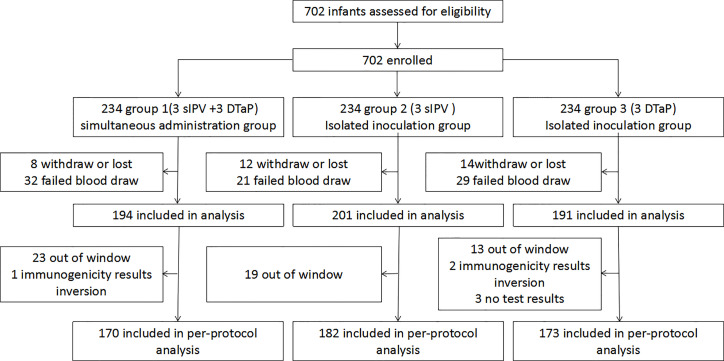
Enrolled subjects and final study population.(group 1 was the co-administration group 1 (3sIPV+ 3DTaP), with the first injection of sIPV at 2 months of age; and the simultaneous inoculation of the sIPV (doses 2 and 3) and DTaP (doses 1 and 2) at 3 and 4 months of age, respectively.Group 2 was the sIPV group (3 sIPV), with 1 dose of the sIPV administered at 2, 3, and 4 months of age, with an interval of 1 month between each dose.Group 3 was the DTaP group (3 DTaP), with 1 dose of DTaP was administered at 3, 4, and 5 months of age, with an interval of 1 month between each dose).

During the study period, a total of 702 subjects were randomly assigned in 1:1:1 ratio to group 1 (3 sIPV +3 DTaP), group 2 (3 sIPV), and group 3 (3 DTaP). Sixty-four subjects in group 1, 52 subjects in group 2, and 61 subjects in group 3 dropped out. There was no statistically significant difference in the drop-out rate among the three groups (P>0.05). Each protocol set consisted of 525 subjects, including 170 in group 1, 182 in group 2, and 173 in group 3 (**Table 1** and **Figure 2**). The baseline demographics were not significantly different between the study groups (**Table 2**).

**Table 1:** Withdrawal of participants during the study process.

Data set	Group 1, n	Group 2, n	Group 3, n	Total, n
Enrolled subjects	234	234	234	702
Complete clinical trials	194	201	191	586
Withdraw during clinical trials	40	33	43	116
Incomplete the vaccination schedule	8	12	14	34
1st dose	0	0	6	6
2st dose	3	6	6	15
3st dose	4	6	2	12
4st dose	1	0	0	1
Failed in blood collection	32	21	29	82
Blood collection before immunization	0	1	0	1
Blood collection after immunization	32	20	29	81
Other deviations from protocol	24	19	15	58
exceed visit window	23	19	13	55
Immunogenicity results inversion	1	0	2	3
Full Analysis Set (FAS)	194	201	189	584
Per-protocol Set (PPS)	170	182	173	525
Safety Set (SS)	234	234	228	696

**Table 2:** Baseline characteristics in per-protocol populations.

	Group 1, n=170	Group 2, n=182	Group 3, n=173	P value
Age(day)				
D (IQR)	72(67-81)	70 (65-78)	72 (66-81)	0.195
Gender				
Male, n (%)	86 (50.59)	100 (54.95)	88 (50.86)	0.654
Female, n (%)	84 (49.41)	82 (45.05)	85 (49.14)	

### Immunogenicity

#### Response to sIPV

**Figure 3:**
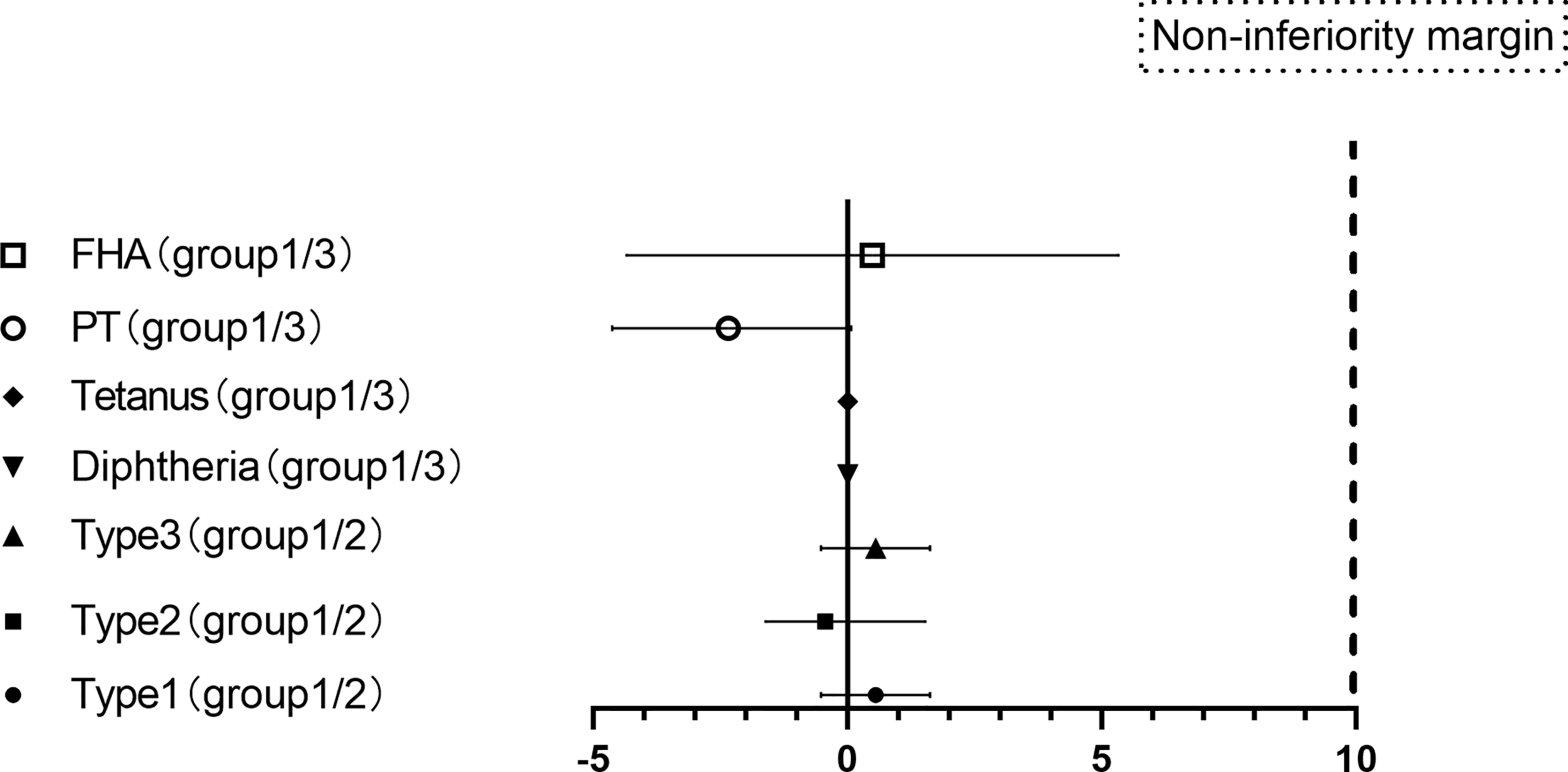
Differences in the proportion of seroconversion to vaccination.(PT: pertussis toxoid, FHA: filamentous hemagglutinin. Differences in the proportion of seroconversion to types 1, 2, and 3 polioviruses were measured between groups 1 and groups 2 with two-sided 95% CIs, and differences in the proportion of seroconversion to PT and FHA of pertussis, diphtheria and tetanus were measured between groups 1 and groups 3 with two-sided 95% CIs.).

The seropositive rates of the three poliovirus types in group 1 and group 2 were not statistically significant prevaccination. After vaccination with the sIPV, the GMTs of subjects for poliovirus types 1, 2, and 3 in group 1 were 369.00, 153.43, and 237.07, respectively, which were lower than those in group 2 (426.47, 170.31, and 269.09) (P> 0.05). There were also no significant differences in the seropositive and seroconversion rates of the three poliovirus types between group 1 and group 2. The non-inferiority rate difference was ≤10% for all three poliovirus types(**Table 3**, **Figure 3**).

**Table 3:** Comparison of immune responses for sIPV+DTaP versus sIPV alone.

Serotype	Parameters of immunogenicity		Group 1, n=170	Group 2, n=182	P value
Type 1	Seropositive, n (%),(95%CI)	Pre-vaccination	41(24.12), (17.9-31.26)	50(27.47), (21.13-34.57)	0.473
		Post-vaccination	100 (97.85-100)	99.45 (96.98-99.99)	1.000
	Seroconversion(%)(95%CI)	Post-vaccination	95.29 (90.94-97.95)	95.05 (90.82-97.71)	0.917
	GMT (95%CI)	Pre-vaccination	5.55 (5.01-6.14)	5.68 (5.17-6.25)	0.504
		Post-vaccination	369.00 (303.48-448.65)	426.47 (355.68-511.34)	0.157
Type 2	Seropositive, n (%),(95%CI)	Pre-vaccination	46(27.06), (20.54-34.39)	47(25.82), (19.63-32.82)	0.793
		Post-vaccination	99.41 (96.77-99.99)	99.45 (96.98-99.99)	0.793
	Seroconversion(%)(95%CI)	Post-vaccination	96.47 (92.48-98.69)	92.31 (87.43-95.73)	0.092
	GMT (95%CI)	Pre-vaccination	5.32 (4.92-5.76)	5.56 (5.06-6.11)	0.925
		Post-vaccination	153.43 (133.53-176.3)	170.31 (149.47-194.06)	0.925
Type 3	Seropositive, n (%),(95%CI)	Pre-vaccination	17(10.00), (5.93-15.53)	16(8.79), (5.11-13.88)	0.697
		Post-vaccination	100 (97.85-100)	98.90 (96.09-99.87)	0.697
	Seroconversion(%)(95%CI)	Post-vaccination	100 (97.85-100)	98.90 (96.09-99.87)	0.697
	GMT (95%CI)	Pre-vaccination	4.73 (4.31-5.2)	4.34 (4.16-4.53)	0.599
		Post-vaccination	237.07 (203.93-275.58)	269.09 (231.37-312.96)	0.276

#### Response to DTaP

Groups 1 and 3 also showed similar responses for all vaccine antigens except anti-FHA [97.65 (94.09-99.36) vs. 100 (97.89-100)], and the non-inferiority criterion of the rate difference was ≤10% for diphtheria, tetanus, PT and FHA. After vaccination with the DTaP, the GMTs of subjects for diphtheria, tetanus, PT and FHA were similar between group 1 and group 3. There was no significant difference in the GMT increase for the following: diphtheria (1.87 versus 1.83, p = 0.276), tetanus (4.70 versus 4.81, p = 0.511), PT (49.79 versus 51.76, p = 0.596) and FHA (86.11 versus 87.00, p = 0.831) (**Table 4** and **Figure 3**).

**Table 4:** Comparison of immune responses for sIPV+DTaP versus DTaP alone.

Antigen	Parameters of immunogenicity		Group 1, n=170	Group 3, n=173	P value
Diphtheria	Seropositive, n (%),(95%CI)	Pre-vaccination	3 (1.76), (0.37-5.07)	10 (5.78), (2.81-10.37)	0.040
		Post-vaccination	100 (97.85-100)	100 (97.89-100)	1.000
	Seroconversion(%)(95%CI)	Post-vaccination	100 (97.85-100)	100 (97.89-100)	1.000
	GMT (95%CI)	Pre-vaccination	0.03 (0.02-0.03)	0.03 (0.03-0.03)	0.280
		Post-vaccination	1.87 (1.71-2.04)	1.83 (1.69-1.99)	0.276
tetanus	Seropositive, n (%),(95%CI)	Pre-vaccination	11(6.47), (3.27-11.28)	9(5.20), (2.41-9.65)	0.616
		Post-vaccination	100 (97.85-100)	100 (97.89-100)	1.000
	Seroconversion(%)(95%CI)	Post-vaccination	100 (97.85-100)	100 (97.89-100)	1.000
	GMT (95%CI)	Pre-vaccination	0.03 (0.02-0.03)	0.02 (0.02-0.02)	0.002
		Post-vaccination	4.70 (4.38-5.03)	4.81 (4.52-5.13)	0.511
PT	Seropositive, n (%),(95%CI)	Pre-vaccination	4(2.35), (0.64-5.91)	0(0), (0-2.11)	0.017
		Post-vaccination	97.06 (93.27-99.04)	94.80 (90.35-97.59)	0.290
	Seroconversion(%)(95%CI)	Post-vaccination	94.71 (90.19-97.55)	94.22 (89.63-97.19)	0.844
	GMT (95%CI)	Pre-vaccination	2.25 (1.96-2.58)	1.84 (1.63-2.07)	0.054
		Post-vaccination	49.79 (46.28-53.55)	51.76 (47.77-56.08)	0.596
FHA	Seropositive, n (%),(95%CI)	Pre-vaccination	4(2.35), (2.35)	2(1.16), (0.14-4.11)	0.394
		Post-vaccination	100 (97.85-100)	100 (97.89-100)	1.000
	Seroconversion(%)(95%CI)	Post-vaccination	97.65 (94.09-99.36)	100 (97.89-100)	0.017
	GMT (95%CI)	Pre-vaccination	3.23 (2.88-3.62)	2.79 (2.48-3.14)	0.064
		Post-vaccination	86.11 (80.07-92.6)	87.00 (81.81-92.52)	0.831

### Safety assessments

**Figure 4:**
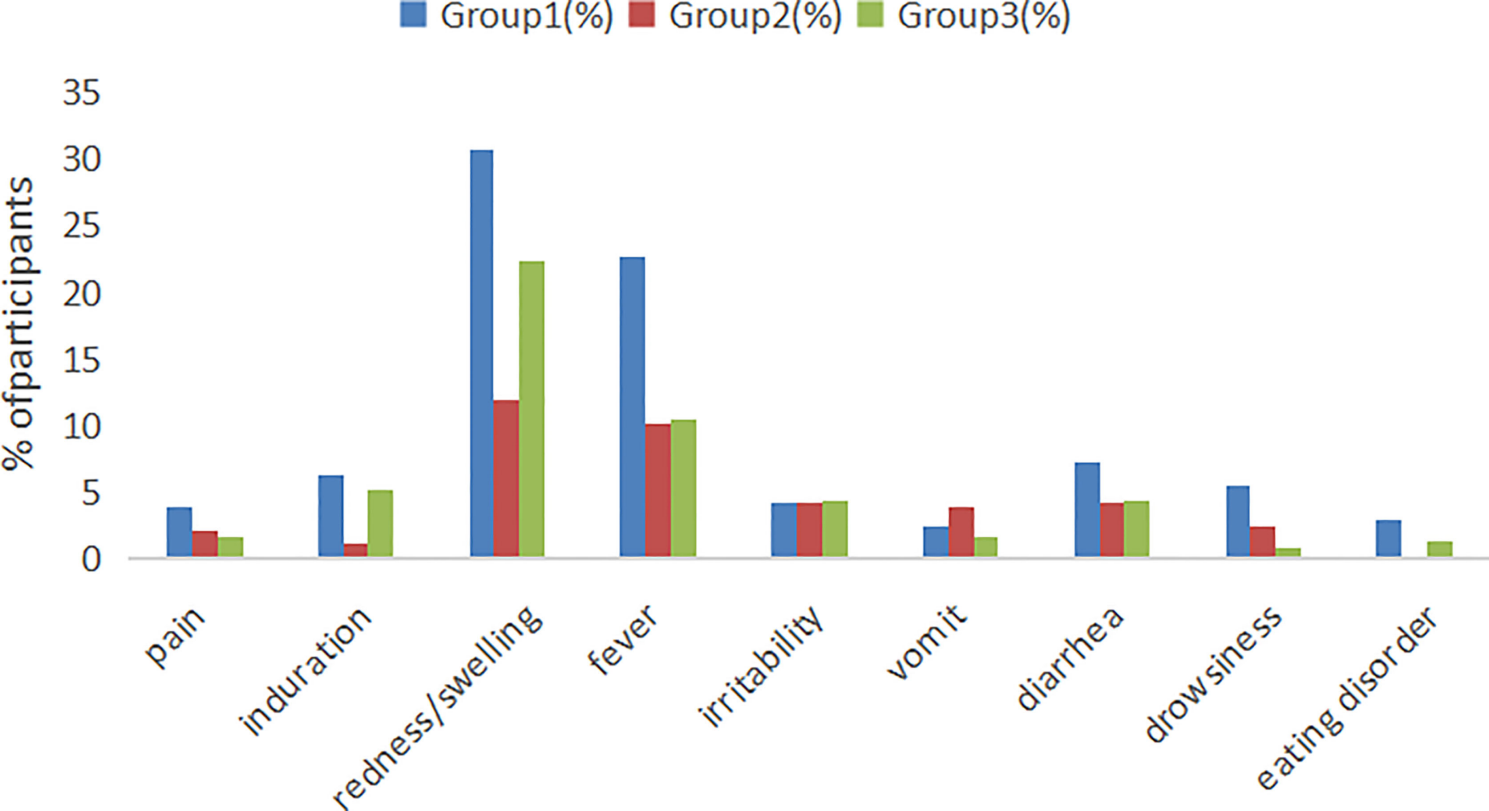
Local and systemic AEs incidence among recipients within 7 days after vaccination. Group 1: (3 sIPV+ 3 DTaP), Group 2: (3 sIPV) and Group 3: (3 DTaP). %: percentage of participants.

According to the research protocol, vaccination with the sIPV and DTaP overlapped at 3 and 4 months of age, which means that the subjects’ 2nd and 3rd vaccinations overlapped. Overall, the sIPV administered concomitantly with the DTaP was well tolerated. **Figure 4** showed that the redness and swelling at the injection site were the most common AEs reported, followed by fever. However, the incidence rates of local AEs, induration, redness and fever reported among groups were significantly different after the third dose (P < 0.05). Further pairwise comparisons showed that there were statistically significant differences between group 1 and group 2 in the incidence rates of local AEs, redness and fever (P < 0.05). The incidence rates of local AEs, redness and fever in group 1 were higher than that in group 2(16.30% vs. 4.50%, 15.42% vs. 4.50%, 5.29% vs. 0.90%) (**Table 5**). There was no significant difference in the severity of AEs among the groups, except for local AEs (P=0.006) and redness (P=0.006), and most AEs were mild or moderate (**Table 6**). After completion of the immunization program, 5 cases of SAEs occurred in the 3 groups, including 1 case requiring hospitalization due to pneumonia, 1 case requiring hospitalization due to influenza, and 3 cases requiring hospitalization due to pneumonia/bronchiolitis/bacterial enteritis in group 1, group 2 and group 3, respectively. Most local and systemic events were mild or moderate, and all 5 cases were determined by experts to be unrelated to the vaccines.

**Table 5:** Local and systemic AEs incidence among recipients within 7 days after vaccination.

AEs	Overall (four doses)			P value	Overall (second dose)			P value	Overall (third dose)			P value
	Group1 n(%)	Group2n(%)	Group3n(%)		Group1n(%)	Group2n(%)	Group3n(%)		Group1n(%)	Group2n(%)	Group3n(%)	
**Local AEs**	70 (29.91)	29 (12.39)	50 (21.93)	<0.01	21 (9.09)	14 (6.14)	14 (6.14)	0.365	37 (16.30)	10 (4.50)	29 (13.06)	<0.01
pain	9 (3.85)	5 (2.14)	4 (1.75)	0.318	4 (1.73)	3 (1.32)	2 (0.88)	0.914	2 (0.88)	0 (0.00)	2 (0.90)	0.554
induration	15 (6.41)	3 (1.28)	12 (5.26)	0.017	2 (0.87)	2 (0.88)	5 (2.19)	0.487	6 (2.64)	1 (0.45)	9 (4.05)	0.043
redness	64 (27.35)	27 (11.54)	48 (21.05)	<0.01	18 (7.79)	14 (6.14)	14 (6.14)	0.715	35 (15.42)	10 (4.50)	27 (12.16)	0.001
swelling	8 (3.42)	1 (0.43)	3 (1.32)	0.044	1 (0.43)	1 (0.44)	1 (0.44)	1.000	4 (1.76)	0 (0.00)	1 (0.45)	0.134
rash	1 (0.43)	0 (0.00)	1 (0.44)	0.774	1 (0.43)	0 (0.00)	0 (0.00)	1.000	0 (0.00)	0 (0.00)	0 (0.00)	–
skin reactions	2 (0.85)	0 (0.00)	0 (0.00)	0.332	1 (0.43)	0 (0.00)	0 (0.00)	1.000	0 (0.00)	0 (0.00)	0 (0.00)	–
**Systemic AEs**	70 (29.91)	44 (18.80)	36 (15.79)	<0.01	26 (11.26)	20 (8.77)	17 (7.46)	0.358	17 (7.49)	8 (3.60)	20 (9.01)	0.063
fever	53 (22.65)	24 (10.26)	24 (10.53)	<0.01	21 (9.09)	9 (3.95)	13 (5.70)	0.069	12 (5.29)	2 (0.90)	12 (5.41)	0.019
irritability	10 (4.27)	10 (4.27)	10 (4.39)	0.998	5 (2.16)	6 (2.63)	6 (2.63)	0.933	1 (0.44)	3 (1.35)	6 (2.70)	0.117
vomit	6 (2.56)	9 (3.85)	4 (1.75)	0.379	2 (0.87)	2 (0.88)	3 (1.32)	0.902	1 (0.44)	1 (0.45)	2 (0.90)	0.849
diarrhea	17 (7.26)	10 (4.27)	10 (4.39)	0.264	5 (2.16)	4 (1.75)	3 (1.32)	0.934	2 (0.88)	2 (0.90)	3 (1.35)	0.902
drowsiness	13 (5.56)	6 (2.56)	2 (0.88)	0.012	3 (1.30)	2 (0.88)	1 (0.44)	0.875	3 (1.32)	1 (0.45)	1 (0.45)	0.627
eating disorder	7 (2.99)	0 (0.00)	3 (1.32)	0.017	2 (0.87)	0 (0.00)	1 (0.44)	0.777	3 (1.32)	0 (0.00)	1 (0.45)	0.331
Allergic	2 (0.85)	2 (0.85)	2 (0.88)	1.000	0 (0.00)	0 (0.00)	0 (0.00)	–	1 (0.44)	0 (0.00)	2 (0.90)	0.550
**Others**	19 (8.12)	15 (6.41)	22 (9.65)	0.440	3 (1.30)	4 (1.75)	6 (2.63)	0.537	6 (2.64)	8 (3.60)	13 (5.86)	0.207

**Table 6:** Severity of adverse events among different groups.

AEs	Group 1 (n=234)			Group 2 (n=234)			Group 3 (n=234)			P value
	Mild,n(%)	Moderate,n(%)	Severe,n(%)	Mild,n(%)	Moderate,n(%)	Severe,n(%)	Mild,n(%)	Moderate,n(%)	Severe,n(%)	
Local AEs	22 (9.4)	39 (16.67)	10 (4.39)	21 (8.97)	5 (2.14)	3 (1.32)	16 (6.84)	25 (10.68)	9 (3.95)	0.006
pain	7 (2.99)	2 (0.85)	0 (0)	4 (1.71)	1 (0.43)	0 (0)	3 (1.28)	1 (0.43)	0 (0)	0.992
induration	5 (2.14)	8 (3.42)	2 (0.88)	2 (0.85)	0 (0)	1 (0.44)	6 (2.56)	5 (2.14)	1 (0.44)	0.721
redness	20 (8.55)	35 (14.96)	9 (3.95)	20 (8.55)	4 (1.71)	3 (1.32)	15 (6.41)	24 (10.26)	9 (3.95)	0.006
swelling	2 (0.85)	4 (1.71)	2 (0.88)	0 (0)	0 (0)	1 (0.44)	1 (0.43)	0 (0)	2 (0.88)	0.485
rash	1 (0.43)	1 (0.43)	0 (0)	0 (0)	0 (0)	0 (0)	1 (0.43)	0 (0)	0 (0)	0.540
skin reactions	2 (0.85)	0 (0)	0 (0)	0 (0)	0 (0)	0 (0)	0 (0)	0 (0)	0 (0)	–
Systemic AEs	37 (15.81)	37 (15.81)	7 (3.07)	36 (15.38)	17 (7.26)	2 (0.88)	26 (11.11)	19 (8.12)	2 (0.88)	0.115
fever	34 (14.53)	25 (10.68)	2 (0.88)	20 (8.55)	9 (3.85)	1 (0.44)	19 (8.12)	10 (4.27)	2 (0.88)	0.720
irritability	10 (4.27)	0 (0)	0 (0)	10 (4.27)	0 (0)	0 (0)	10 (4.27)	0 (0)	0 (0)	1.000
vomit	5 (2.14)	0 (0)	1 (0.44)	7 (2.99)	4 (1.71)	0 (0)	4 (1.71)	1 (0.43)	0 (0)	0.839
diarrhea	7 (2.99)	14 (5.98)	5 (2.19)	8 (3.42)	5 (2.14)	1 (0.44)	6 (2.56)	12 (5.13)	1 (0.44)	0.204
drowsiness	13 (5.56)	0 (0)	0 (0)	5 (2.14)	1 (0.43)	0 (0)	2 (0.85)	0 (0)	0 (0)	0.843
eating disorder	5 (2.14)	2 (0.85)	0 (0)	0 (0)	0 (0)	0 (0)	3 (1.28)	0 (0)	0 (0)	0.494
Allergic reaction	1 (0.43)	1 (0.43)	0 (0)	1 (0.43)	1 (0.43)	0 (0)	2 (0.85)	0 (0)	0 (0)	0.651
Others	18 (7.69)	27 (11.54)	14(6.14)	15 (6.41)	22 (9.4)	8 (3.51)	22 (9.4)	32 (13.68)	6 (2.63)	0.406

## Discussion

To prevent common infectious diseases among children, the health divisions of each country recommend that children should be vaccinated according to the vaccination schedule. Due to improvements in vaccine research and development technology, an increasing number of vaccines have been produced. In many cases, these vaccines will overlap in vaccination schedules. However, an indiscriminate combination of antigens may affect the immunogenicity of individual components. For example, Haemophilus influenza type B (Hib) vaccines reduce the immunogenicity of some vaccine components (12, 13). In this study, we found that seroconversion of antibodies with sIPV vaccine and DTaP vaccine was not disturbed by co-administration, and the high seroconversion rates and high antibody concentrations found in the co-administered group provided reassurance that Infants were protected from these several diseases, whether in the concomitant or separated groups.These data supported the introduction of a strategy of co-administration of sIPV and DTaP vaccine into routine childhood immunization programs.The DTaP-IPV combination vaccine has been demonstrated to have good immunogenicity and safety in previous studies (14, 15). Combination vaccines are not widely available in low- and middle-income countries due to price factors, and co-administration of the two vaccines may be particularly useful in areas with scarce resources as it would avoid additional clinic visit and inconvenience.

The incidence of local and systemic AEs in the co-administration group was slightly higher than that in the separate group. DTaP has been used in China for decades, while the sIPV from Beijing Institute of Biological Products was approved for marketing in 2017 (16), and pre-certified by WHO in 2022 (17), the two vaccines were considered safe. Passive post-marketing surveillance of DTaP-IPV/Hib vaccination in Guangzhou of China from 2011 to 2017 found no safety concerns, with 728 Adverse Events Following Immunization(AEFI)cases reported per million doses (18). Redness and swelling at the injection site were the most common AEs reported, followed by fever, which was consistent with previous studies (19, 20). This study showed that the incidence of redness and swelling at the injection site of group 1 (30.77%) and group 3 (22.37%) with DTaP vaccine was significantly higher than that of group 2 (11.97%), and the incidence of local reactions increased with the increase of the number of doses (**Table 5**). The reports of injection site redness and swelling in this study are consistent with observations from an earlier Korean study that assessed swelling response at the injection site after DTaP-IPV combined or separate administration, in which 27.0% of DTaP-IPV combination subjects, 17.6% of subjects with DTaP and salk IPV stand alone reported redness and swelling at the injection site (20). Several studies had demonstrated that pertussis toxin, diphtheria toxoid and aluminum content in the vaccine, high antibody titers to diphtheria, tetanus or pertussis toxin before vaccination and a Th2 orientation of cytokine production may lead to enhanced local response (21, 22). In addition, the upper arm deltoid muscle was selected as the injection site of the vaccine, which also leads to the increased incidence of local reactions to a certain extent in our study, some studies had confirmed this (23, 24). At the same time, subcutaneous injection and insufficient shaking before vaccination may also increase the risk of redness, swelling and induration at the injection site (25).The fever collected in the combined vaccination group was 22.65%, which was higher than that in the separate vaccination group. Compared with some foreign studies (26, 27), the incidence was higher, but lower than a combined vaccine (DTPa-IPV/Hib) clinical trail in Chinese children (15). In fact, the study was started in December 2019, and subjects were at higher risk for fever (such as upper respiratory tract infections) due to cold weather. Fortunately, our study showed that the safety of simultaneous administration of DTaP and sIPV was non-inferior to single vaccination, and the simultaneous administration was completely acceptable. Most of the AEs were usually mild and moderate, and all 5 cases SAEs were determined by experts to be unrelated to the vaccine.

Differences in the immunogenicity and safety of the DTaP-IPV combination vaccine versus the separate DTaP and IPV vaccine were evaluated in a randomized study of 37-week-old infants in South Korea in 2009-2010 (20). The researchers showed that the seroconversion rate of the DTaP-IPV combination vaccine group was not lower than that of the single vaccination group one month after vaccination, and even the seroconversion rate of anti-PT and anti-FHA was higher in the DTaP-IPV combination vaccine group (96.6 [93.1-98.6] vs 94.4 [90.4-97.1], 92.2 [87.6-95.5] vs 78.4 [72.3-83.7]), and the GMC of the three types of poliovirus, tetanus and FHA of pertussis antibody titers in the DTaP-IPV combined vaccine group were higher than those in the single administration group.Another Chinese study similar to South Korea also showed that whether inoculated with DTAP-IPV combination vaccine at 2/3/4/5 months of age or 3/4/5 months of age (28), the seroconversion rate of antibodies was not lower, and the GMC of antibody titers except diphtheria were much higher than that in the single-administration group. Domestic DTaP and sIPV were used in our study, the seroconversion rate was consistent with that of imported vaccines, but the GMC especially the antibody titers of 3 types of polio were lower.The difference may be caused by the characteristics of Sabin Strain IPV and Salk Strain IPV (29–31).

The introduction of co-administration strategy into routine infant immunization is under consideration in China and some less developed countries, but information with the immunogenicity and safety of co-administration needs to be provided due to the high rate of seroconversion required to protect the population. The non-inferiority and safety of combined DTaP and sIPV shown in this randomized trial supports co-administration in infants, and this finding has important implications for controlling the epidemic of infectious diseases in China and even globally.

The study began on work in August 2019, and the preliminary progress was smooth. However, in December 2019, the sudden outbreak of COVID - 19, it had affected the progress of clinical trials. Moreover, the study was conducted in an Asian country, and the results may not be applicable to other ethnic groups.

Overall, we found equal rates of seroconversion when DTaP was administered alone and simultaneously with sIPV, and no safety concerns were identified with either vaccine used in this study. Administering both vaccines at the same time reduces the time and effort invested by staff and guardians, and it could increase vaccination coverage and protect more infants from morbidity and mortality from these related diseases.

## Data availability statement

The raw data supporting the conclusions of this article will be made available by the authors, without undue reservation.

## Ethics statement

The studies involving human participants were reviewed and approved by The clinical trial was approved by the ethics committees of the Jiangsu Province Center for Disease Control and Prevention. Written informed consent to participate in this study was provided by the participants’ legal guardian/next of kin.

## Author contributions

YX, YH X and FT were responsible for data curation. XS and ZW were responsible for formal analyses. XS and XY were responsible for study supervision. XS was responsible for writing the original draft of the manuscript. BW, XZ and HC were responsible for reviewing and editing the manuscript. All authors contributed to the article and approved the submitted version.

## Funding

This work was supported by China National Biotec Group Company Limited. The funder was not involved in the study design, collection, analysis, interpretation of data, the writing of this article or the decision to submit it for publication.

## Acknowledgments

We thank the staff of the sheyang, si country and jiangyou Center for Disease Control and Prevention, who spent numerous hours and effort working with us in obtaining, verifying, and cleaning up the data used in this study.

## Conflict of interest

YH X, HC and XY are employees of China National Biotec Group Company Limited.

The remaining authors declare that the research was conducted in the absence of any commercial or financial relationships that could be construed as a potential conflict of interest.

## Publisher’s note

All claims expressed in this article are solely those of the authors and do not necessarily represent those of their affiliated organizations, or those of the publisher, the editors and the reviewers. Any product that may be evaluated in this article, or claim that may be made by its manufacturer, is not guaranteed or endorsed by the publisher.
